# Barley Sprouts Extract Attenuates Alcoholic Fatty Liver Injury in Mice by Reducing Inflammatory Response

**DOI:** 10.3390/nu8070440

**Published:** 2016-07-21

**Authors:** Yun-Hee Lee, Joung-Hee Kim, Sou Hyun Kim, Ji Youn Oh, Woo Duck Seo, Kyung-Mi Kim, Jae-Chul Jung, Young-Suk Jung

**Affiliations:** 1 College of Pharmacy, Yonsei University, Incheon 21983, Korea; yunhee.lee@yonsei.ac.kr; 2 Department of Bio Health Science, College of Natural Science, Changwon National University, Changwon 51140, Korea; k9i1h@naver.com; 3 College of Pharmacy, Pusan National University, Busan 46241, Korea; hyunie9808@naver.com (S.H.K.); pooh7282@naver.com (J.Y.O.); 4 Crop Foundation Division, National Institute of Crop Science, Rural Development Administration, Wanju-Gun, Jeollabuk-do 54875, Korea; swd@korea.kr; 5 Life Science Research Institute, Novarex Co., Ltd, Ochang, Cheongwon, Cheongju 28126, Korea; kkm3507@novarex.co.kr (K.-M.K.); jcjung@novarex.co.kr (J.-C.J.)

**Keywords:** barley sprouts, alcohol-induced liver injury, glutathione, TNF-α, inflammation

## Abstract

It has been reported that barley leaves possess beneficial properties such as antioxidant, hypolipidemic, antidepressant, and antidiabetic. Interestingly, barley sprouts contain a high content of saponarin, which showed both anti-inflammatory and antioxidant activities. In this study, we evaluated the effect of barley sprouts on alcohol-induced liver injury mediated by inflammation and oxidative stress. Raw barley sprouts were extracted, and quantitative and qualitative analyses of its components were performed. The mice were fed a liquid alcohol diet with or without barley sprouts for four weeks. Lipopolysaccharide (LPS)-stimulated RAW 264.7 cells were used to study the effect of barley sprouts on inflammation. Alcohol intake for four weeks caused liver injury, evidenced by an increase in serum alanine aminotransferase and aspartate aminotransferase activities and tumor necrosis factor (TNF)-α levels. The accumulation of lipid in the liver was also significantly induced, whereas the glutathione (GSH) level was reduced. Moreover, the inflammation-related gene expression was dramatically increased. All these alcohol-induced changes were effectively prevented by barley sprouts treatment. In particular, pretreatment with barley sprouts significantly blocked inducible nitric oxide synthase (iNOS) and cyclooxygenase (COX)-2 expression in LPS-stimulated RAW 264.7. This study suggests that the protective effect of barley sprouts against alcohol-induced liver injury is potentially attributable to its inhibition of the inflammatory response induced by alcohol.

## 1. Introduction

Fatty liver or hepatic steatosis is defined as the accumulation of triglycerides in the liver, leading to more than 5% of the hepatic cells containing either micro- or macrovesicular lipid droplets. Multiple factors are involved in the induction of fatty liver; however, obesity, diabetes, and dyslipidemia, as well as excessive alcohol drinking, are the most frequent causes of fatty liver. Especially, alcoholic liver disease (ALD), one of the major chronic liver diseases, is characterized by a complex spectrum ranging from simple steatosis to cirrhosis. Although it is highly prevalent and holds a high rank in the causes of death worldwide, preventive and therapeutic approaches have yet to be discovered [1,2].

A well-known mechanism of alcohol-induced hepatotoxicity is its ability to induce inflammatory responses and oxidative stress following free radical formation [3]. The liver cells have various sources of reactive oxygen species (ROS) which are activated by chronic alcohol consumption, leading to an increase in the generation of oxidants [4]. In detail, they are generated by a complex pathway by metabolic enzyme-mediated oxidation, abnormal mitochondrial function, Kupffer cell activation, disruption of lipid metabolism, and cytokine production [3,5,6]. Notably, alcohol consumption increases the permeability of intestinal mucosa and sensitizes Kupffer cells to activation by endotoxins via Toll-like receptor 4 (TLR4). The harmful paracrine effects of Kupffer cell activation include the production of various inflammatory mediators as well as ROS, which contribute to the pathological progression of ALD from simple fat accumulation to steatohepatitis [7,8,9,10]. Indeed, Li et al. [11] evaluated whether steatosis has inflammatory biomarkers using clinical and experimental approaches. In this study, the group with fatty liver showed a significantly higher level of serum tumor necrosis factor (TNF)-α than the control group did, which correlated with the pathological severity of the hepatic lesions [11].

**Figure 1:**
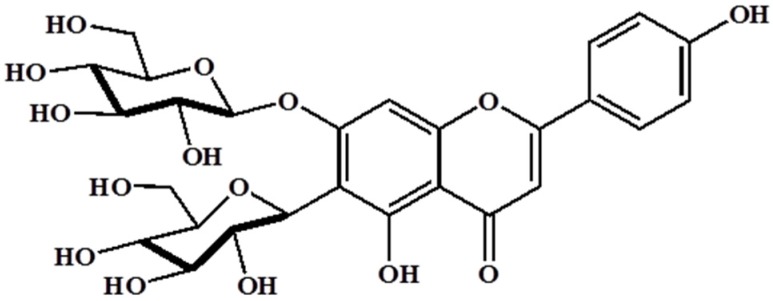
Structure of saponarin, an active compound in barley sprouts extract.

Barley (*Hordeum vulgare* L.) has been used as a food material since ancient times and was one of the first cultivated grains, particularly in Eurasia, as far back as 13,000 years ago. It has also been used as animal fodder, a source of fermentable material for beer and certain distilled beverages, and a component of numerous health foods. Barley sprouts, which are the young leaves of barley harvested approximately 10 days after sowing the seeds, have recently received much attention as a functional food in numerous countries, especially Japan and Korea. It has been reported that barley leaves possess beneficial properties such as antioxidant, hypolipidemic, antidepressant, and antidiabetic [12,13,14]. Interestingly, barley sprouts contain a high content of saponarin (Figure 1), which is a member of the flavonoid family, in addition to policosanol polyphenol series, various minerals, and free amino acids. Among these, saponarin is the major compound in barley sprouts, and it shows both anti-inflammatory and antioxidant activities. LPS-induced inflammation in RAW 264.7 cells was significantly inhibited by treatment with saponarin isolated from barley sprouts, and this effect is mediated by the inhibition of nuclear factor kappa-light-chain-enhancer of activated B cells (NF-κB), extracellular signal-regulated kinase (ERK), and p38 signaling [15]. In addition, saponarin prevented cocaine or paracetamol-induced hepatotoxicity by inducing the hepatic antioxidant capacity [16,17]. Based on these reports, we hypothesized that the extract of barley sprouts could have the potential to ameliorate chronic alcohol-induced liver injury mediated by an inflammatory response and, therefore, we investigated our hypothesis in this study.

## 2. Materials and Methods 

### 2.1. Extraction

**Figure 2:**
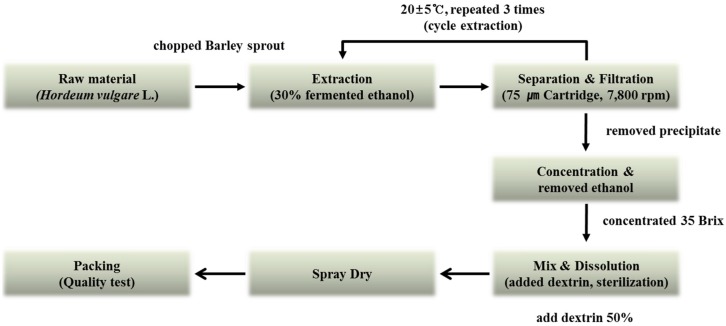
Manufacturing process for production of barley sprouts extract powder.

The barley sprouts were cultivated in Yeonggwang-gun, Jeollanam-do Province, Korea. The original grain of barley used was the saechalssal (hulled barley), and the extract was prepared from the barley sprouts after they had grown to a length of approximately 20 cm. The raw material was provided by Saeddeumwon Co., Ltd. (Yeonggwang, Korea) in 2015 and the extract was produced by Novarex Co., Ltd. (Ochang, Korea). The analysis of the biological component and microbiological test were confirmed by Novarex Co., Ltd. All other chemicals were purchased from Sigma-Aldrich Chemical Corp. (St. Louis, MO, USA) and Wako Pure Chemical Industries (Osaka, Japan). The raw barley sprouts plant material was extracted for 9 h at 25 °C by exposing it to circulating 30% aqueous fermented ethanol. Then, the extract was filtered through a 75-μm cartridge, and the residue was removed by using centrifugation. The supernatant was vacuum-concentrated under reduced pressure (10 atm, 55–58 °C) to attain 35 brix materials. Then, it was blended with dextrin, sterilized at 95 °C for 30 min, followed by spray-drying (liquid temperature, 75–80 °C; blowing temperature, 180 °C; atomizer, 18,000 rpm) to obtain a barley sprouts extract powder. To establish the bulk scale production of the barley sprouts extract we optimized the manufacturing process based on experimental pilot conditions (Figure 2). 

### 2.2. Analysis of Saponarin Using Liquid Chromatography-Tandem Mass Spectrometry (LC-MS/MS)

We performed the material separation using the LUNA C_18_ column (2.0 × 150 mm, 5-μm). Solvent A was water with 0.1% formic acid, and solvent B was acetonitrile with 0.1% formic acid. The gradients of the solvents were as follows: 0 min, 10% B; 1 min, 10% B; 7 min, 70% B; 8.5 min, 70% B; 9 min, 10% B; and 15 min, 10% B. The samples were dissolved in 50% acetonitrile and the injection volume was 5 μL. We used digoxin as the internal standard to quantify saponarin in barley sprouts extract, and the detailed conditions for the liquid chromatography-tandem mass spectrometry (LC-MS/MS) analysis are shown in Table 1.

### 2.3. Animals and Treatments

Male C57BL/6 mice were obtained from Orient Bio (Sungnam, Korea). The use of the animals was in compliance with the guidelines established and approved by the Institutional Animal Care and Use Committee of Pusan National University (PNU-2015-1027). The animals were allowed to acclimate to temperature (22 ± 2 °C)- and humidity (55% ± 5%)-controlled rooms with a 12 h light/dark cycle for one week prior to use. The mice were fed a Lieber–DeCarli liquid alcohol diet (Dyets Inc., Bethlehem, PA, USA) with or without barley sprouts for four weeks. For the control diet, 35% of the energy was derived from fat, 18% from protein, and 47% from carbohydrates, while the alcohol diet contained 35% of energy from fat, 18% from protein, 11% from carbohydrates, and 36% from ethanol (Table 2). The barley sprouts were administered by gavage daily, and silymarin (Sigma-Aldrich Chemical Corp., St. Louis, MO, USA) was treated as the positive control.

### 2.4. Hematological and Histopathological Evaluation of Liver Injury

The serum activities of alanine aminotransferase (ALT) and aspartate aminotransferase (AST) and total triglyceride (TG) levels were determined by using an automated chemistry analyzer (Prestige 24I, Tokyo Boeki Medical System, Tokyo, Japan). The serum concentration of TNF-α was measured by using an enzyme-linked immunosorbent assay (ELISA) using a commercially available kit (R & D Systems, Minneapolis, MN, USA) according to the manufacturer’s instruction. The serum endotoxin level was assessed using the Limulus Amebocyte Lysate chromogenic endotoxin quantitation kit (Thermo Scientific, Sunnyvale, CA, USA). To evaluate the lipid accumulation in the liver tissue, 5-μm cross sections of the left lateral lobe of the liver were sliced, immersed in propylene glycol for 5 min, and then stained with Oil red O. After washing with 85% propylene glycol and distilled water, the sections were counterstained with hematoxylin for 2 min before microscopic examination.

### 2.5. Determination of Hepatic TG Content

The total lipids were extracted from 100 mg of liver tissue using a mixture of chloroform/methanol (2:1, *v/v*). To determine the TGs content of the total lipids, a commercially available enzymatic kit (Sigma-Aldrich Chemical Corp., St. Louis, MO, USA) was used according to the manufacturer’s instruction.

### 2.6. Measurement of Hepatic Glutathione (GSH)

The liver homogenate was prepared by using a 4-fold volume of ice-cold 1 M perchloric acid. After centrifugation at 10,000× *g* for 10 min to remove the denatured protein, the total GSH level in the supernatant was measured by using a high-performance liquid chromatography (HPLC) separation/fluorometric detection method [18].

### 2.7. Cell Culture and Viability Assay

The RAW 264.7 cells were obtained from the American Type Culture Collection (ATCC, Manassas, VA, USA) and were grown in Dulbecco’s modified Eagle’s medium (DMEM) containing 10% fetal bovine serum (FBS), 2 mM glutamine, 100 U/mL penicillin, and 100 μg/mL streptomycin (GenDEPOT, Barker, TX, USA) at 37 °C in a humidified incubator with 5% CO_2_. Cell viability was determined by EZ-CyTox (Daeil Lab Service, Seoul, Korea). After 24 h incubation of cells with barely sprouts extract, EZ-CyTox solution was added to each well and it was measured at a 450 nm. The results were expressed as a percentage compared to the vehicle treated cells.

### 2.8. Determination of Nitric Oxide (NO) Production

After stimulating the cells with 200 ng/mL of LPS (Sigma-Aldrich, St. Louis, MO, USA) for 24 h, the culture medium was collected and assayed for NO production. A 50 μL aliquot of the medium was mixed with 50 μL Griess reagent (1% sulfanilamide in 5% phosphoric acid and 0.1% naphthylethlyenediamene dihydrochloride) and then incubated for 20 min. The absorbance was measured at 540 nm using a microplate reader (Multiskan™ GO microplate spectrophotometer, Thermo Scientific, Sunnyvale, CA, USA). The NO concentration was determined by using a sodium nitrite standard curve.

### 2.9. Western Blotting

Cells were lysed with ice-cold PRO-PREP™ protein extract solution (iNtRON, Sungnam, Gyunggi, Korea) and the protein concentration was determined by using the bicinchoninic acid (BCA) procedure (Thermo Scientific, Sunnyvale, CA, USA). Equal amounts of protein were separated by using sodium dodecyl sulfate-polyacrylamide gel electrophoresis (SDS-PAGE) and then transferred onto a polyvinylidene difluoride (PVDF) membrane (Millipore, Billerica, MA, USA). The membrane was blocked with 5% skim milk in 100 mM Tris-hydrochloride (HCl, pH 7.5), 150 mM sodium chloride (NaCl), and 0.2% Tween-20 (TBST) for 1 h at room temperature. The membranes were incubated with TBST containing 5% milk and the primary antibodies against anti-inducible nitric oxide synthase (iNOS), anti-cyclooxygenase (COX)-2, and β-actin (Santa Cruz Biotechnology, Santa Cruz, CA, USA). After washing with TBST, the blot was incubated with the appropriate horseradish peroxidase (HRP)-conjugated secondary antibodies. The antigen was detected by using a Western Bright enhanced chemiluminescence (ECL) HRP substrate kit (Advansta, Menlo Park, CA, USA).

### 2.10. Real-Time Reverse Transcription-Polymerase Chain Reaction (RT-PCR)

The total RNA was isolated from liver tissue and cells using the RNeasy kit (Qiagen, Valencia, CA, USA). Then, the cDNA was synthesized by using the iScript™ cDNA Synthesis system (Bio-Rad, Hercules, CA, USA). The real-time RT-PCR was performed by using the SensiFAST SYBR qPCR mix (Bioline, London, UK) according to the manufacturer’s protocol. The relative values of gene expression were normalized to 18S ribosomal RNA. The primer sequences and full gene names are provided in Table 3.

### 2.11. Statistical Analysis

All the results are expressed as mean ± standard deviation (SD) and were analyzed by using a one-way analysis of variance (ANOVA) followed by the Newman–Keuls multiple range test (parametric). The acceptable level of significance was established at *p* < 0.05.

## 3. Results

### 3.1. Analysis of Barley Sprouts Extract Composition 

**Figure 3:**
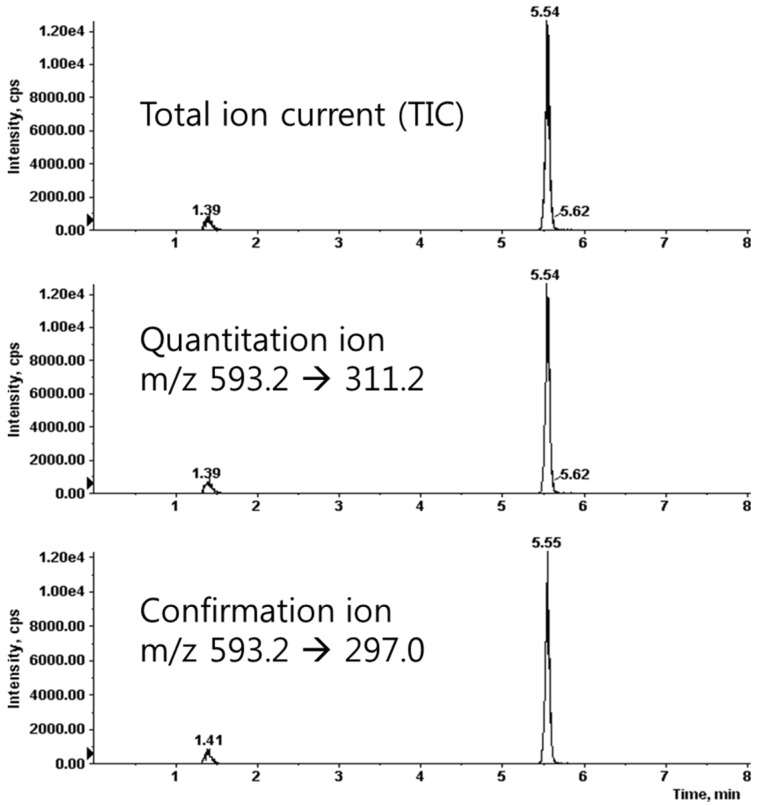
Liquid chromatography-tandem mass spectrometry (LC-MS/MS) spectrum of saponrin.

We prepared the calibration curves for saponarin according to a concentration-dependent electrospray ionization-mass spectrometer (ESI-MS) method. We found that the correlation coefficient (*r*^2^) value was 0.9997, which showed good linearity of the calibration curves. The limits of detection and quantification were 2.32 and 7.03 ng/mL, respectively. The condition of the multiple-reaction-monitoring (MRM) mode was m/z 593.2 (precursor ion) → 311.2 (product ion) for saponarin (Figure 3). For the quantitative analysis, we used the calibration curves to calculate the ratios of compounds in the analyzed material to their respective standards. We diluted the barley sprouts extract 1/5 to ensure that the concentration of its components was within the quantitative range of the calibration curves and then multiplied the obtained concentration by 5 (the dilution factor). The quantitative result of saponarin was 14.74 ± 0.27 μg/mg in the barley sprouts extract. 

### 3.2. Preventive Effect of Barley Sprouts on Alcohol-Induced Liver Injury

**Figure 4:**
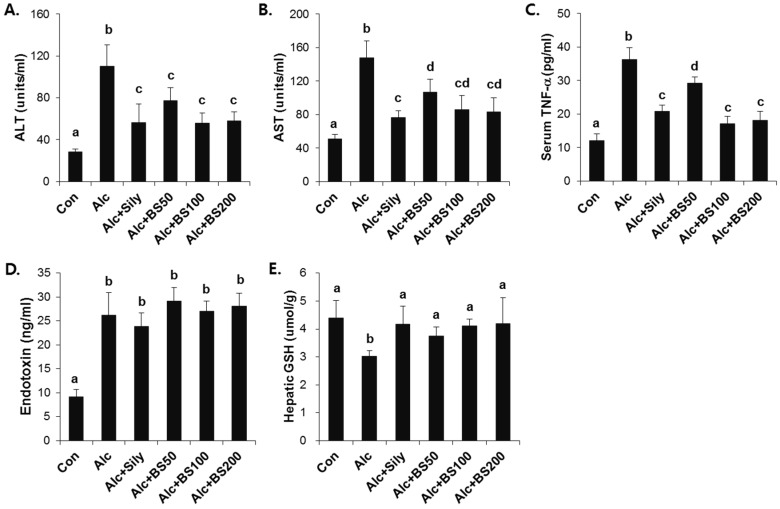
Dose-dependent effect of barley sprouts extract on alcohol-induced liver injury. Serum (**A**) ALT and (**B**) AST activities; (**C**) TNF-α; and (**D**) endotoxin level in the serum; and (**E**) GSH concentration in the liver. Each value is mean ± standard deviation (SD) of six mice. Values with different letters are significantly different by analysis of variance (ANOVA) followed by Newman–Keuls multiple range test (*p* < 0.05). ALT, alanine aminotransferase; AST, aspartate aminotransferase; TNF, tumor necrosis factor; Con, control diet fed mice; Alc, alcohol diet fed mice; Alc + Sily, Alc fed mice treated with 100 mg/kg silymarin; Alc + BS50, Alc fed mice treated with 50 mg/kg barley sprouts extract; Alc + BS100, Alc fed mice treated with 100 mg/kg barley sprouts extract; Alc + BS200, Alc fed mice treated with 200 mg/kg barley sprouts extract.

**Figure 5:**
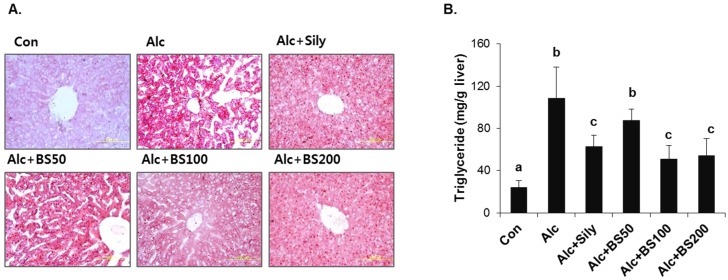
Lipid accumulation in liver of mice treated with alcohol with or without barley sprouts extract. (**A**) Oil red O staining of liver at same magnification (400×) and (**B**) liver triglyceride (TG). Each value is mean ± standard deviation (SD) of six mice. Values with different letters are significantly different by analysis of variance (ANOVA) followed by Newman–Keuls multiple range test (*p* < 0.05). Con, control diet fed mice; Alc, alcohol diet fed mice; Alc + Sily, Alc fed mice treated with 100 mg/kg silymarin; Alc + BS50, Alc fed mice treated with 50 mg/kg barley sprouts extract; Alc + BS100, Alc fed mice treated with 100 mg/kg barley sprouts extract; Alc + BS200, Alc fed mice treated with 200 mg/kg barley sprouts extract.

To test the effect of barley sprouts on the alcohol-induced liver injury, a study was performed in mice fed a standard Lieber–DeCarli liquid diet supplemented with ethanol for four weeks to determine the potential dose-dependency of the extract. Different doses of the barley sprouts extract ranging from 50 to 200 mg/kg body weight were orally administered daily from the beginning of the liquid diet. We compared the effect of the barley sprouts extract on the alcoholic liver injury with that of silymarin (100 mg/kg body weight), a well-known compound that alleviates alcohol-induced liver injury, as a positive control. The serum ALT and AST activities and TNF-α level in the alcohol-fed mice were all significantly higher than those in the control diet-fed mice were (Figure 4A–C). Whereas the hepatic GSH concentration in the alcohol-treated mice was significantly decreased compared with that of the control mice (Figure 4E). All these alcohol-induced changes were prevented significantly by supplementation with barley sprouts extract at doses exceeding 100 mg/kg. Interestingly, the increased serum level of endotoxin in the alcohol-fed mice was not changed by treatment of barley sprouts extract (Figure 4D). The lipid content of the liver of the alcohol-treated mice showed dramatic accumulation when examined by using both the oil red O staining (Figure 5A) and triglyceride content determination (Figure 5B). Furthermore, supplementation with more than 100 mg/kg of barley sprouts extract significantly reversed the hepatic lipid accumulation (Figure 5). These results were comparable with those obtained with silymarin treatment, indicating that the barley sprouts extract could be a promising candidate to protect against liver injury induced by chronic alcohol ingestion. 

### 3.3. Inhibitory Effect of Barley Sprouts Extract on Inflammatory Response-Related Gene Expression in Liver of Alcohol-Treated Mice

**Figure 6:**
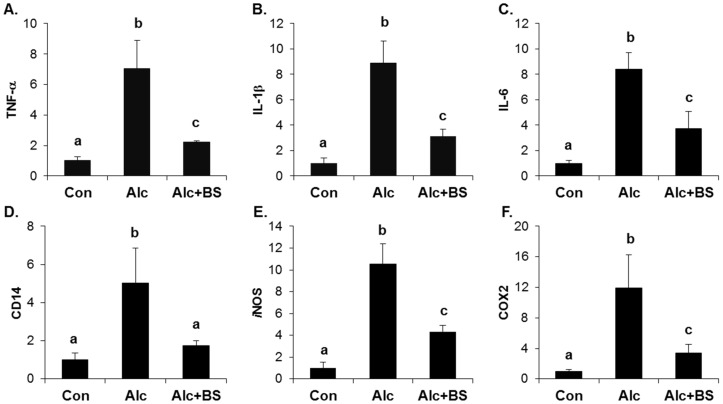
Effect of barley sprouts extracts on inflammation-related gene expression in liver of alcohol-treated mice. mRNA expression of liver (**A**) TNF-α; (**B**) IL-1β; (**C**) IL-6; (**D**) CD14; (**E**) iNOS; and (**F**) COX2 using real-time RT-PCR. Each value is mean ± standard deviation (SD) of six mice. Values with different letters are significantly different by analysis of variance (ANOVA) followed by Newman–Keuls multiple range test (*p* < 0.05). TNF, tumor necrosis factor; IL, interleukin; CD, cluster of differentiation; iNOS, inducible nitric oxide synthase; COX, cyclooxygenase; RT-PCR, reverse transcription-polymerase chain reaction; Con, control diet fed mice; Alc, alcohol diet fed mice; Alc + BS, Alc fed mice with 200 mg/kg barley sprouts extract.

The importance of the inflammatory response in alcoholic liver injury has been suggested. Increased circulating endotoxin activates Kupffer cells, which are the resident liver macrophage, and leads to the induction of cytokines, chemokines, and ROS. Here, we determined the expression level of inflammation-related genes using qPCR. The hepatic mRNA expression of TNF-α, interleukin (IL)-1β, IL-6, a cluster of differentiation (CD) 14, iNOS, and COX2 was induced significantly in the liver of the alcohol-treated mice (Figure 6). Supplementation with the barley sprouts extract for the entire alcohol consumption period effectively inhibited the increase of all the mRNAs determined in this experiment (Figure 6). 

### 3.4. Anti-Inflammatory Effect of Barley Sprouts Extract in LPS-Activated Raw 264.7 Cells

**Figure 7:**
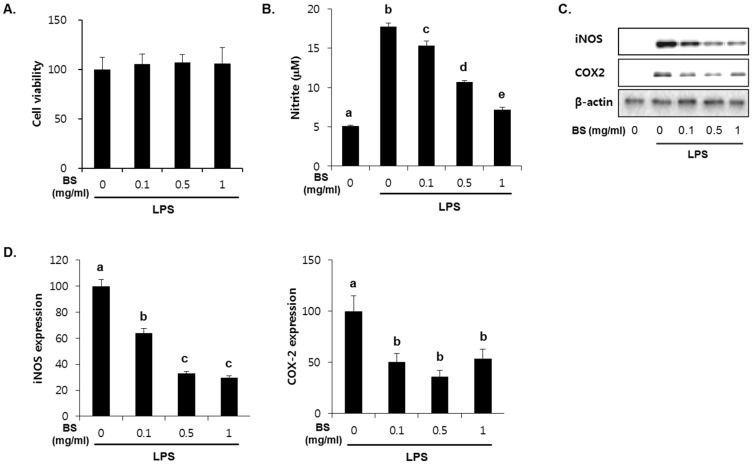
Dose-dependent effect of barley sprouts extract on nitric oxide (NO) generation and protein expression of inducible nitric oxide synthase (iNOS) and cyclooxygenase (COX)-2 in lipopolysaccharide (LPS)-stimulated Raw 264.7 cells. (**A**) Cell viability was determined after treatment with barley sprouts extract for 24 h. Cells were pretreated with barley sprouts extract for 1 h before treatment with 200 ng/mL LPS for 24 h; (**B**) NO generation in medium and (**C**) protein expression of iNOS and COX2 in whole cell lysates; (**D**) Quantitative analysis of blots. Each value is mean ± standard deviation (SD) of triplicates in three independent experiments. Values with different letters are significantly different by analysis of variance (ANOVA) followed by Newman–Keuls multiple range test (*p* < 0.05). BS, barley sprouts extract; iNOS, inducible nitric oxide synthase; COX, cyclooxygenase.

**Figure 8:**
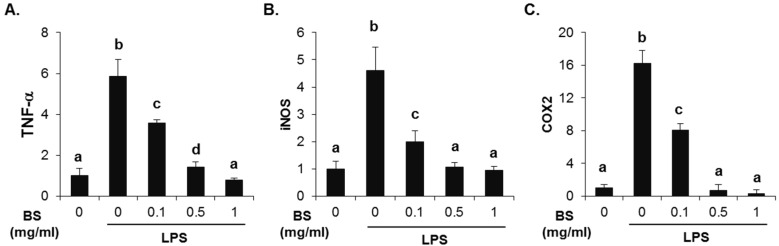
Effect of barley sprouts extract on inflammation-related gene expression in lipopolysaccharide (LPS)-stimulated Raw 264.7 cells. Cells were pretreated with barley sprouts extract for 1 h before treatment with 200 ng/mL LPS for 6 h. mRNA expression of (**A**) TNF-α; (**B**) iNOS; and (**C**) COX2 using real-time RT-PCR. Each value is mean ± standard deviation (SD) of triplicates in three independent experiments. Values with different letters are significantly different by analysis of variance (ANOVA) followed by Newman–Keuls multiple range test (*p* < 0.05). BS, barley sprouts extract; iNOS, inducible nitric oxide synthase; COX, cyclooxygenase; RT-PCR, reverse transcription-polymerase chain reaction.

To investigate whether the barley sprouts extract regulates NO production, cells were pretreated with the extract for 1 h before treatment with LPS for 24 h. As shown in Figure 7A, no cytotoxic effects of barley sprouts extract were observed. Treatment with LPS significantly upregulated the nitrite production (17.7 ± 0.4 μM) compared to that of the untreated control (5.1 ± 0.1 μM, Figure 7B). However, RAW 264.7 cells pretreated with barley sprouts extract displayed a marked decrease in nitrite in a dose-dependent manner after stimulation with LPS. Next, we investigated whether barley sprouts extract regulates iNOS protein expression. Consistent with the suppression of nitrite production, the barley sprouts extract dose-dependently inhibited iNOS protein expression (Figure 7C,D). In addition, the protein expression of COX2 also showed the same pattern as that of iNOS (Figure 7C,D). These data indicate that the barley sprouts extract attenuated the upregulation of LPS-induced COX2 and iNOS expression. Next, we determined the effect of barley sprouts extract on the mRNA expression of TNF-α, iNOS, and COX2 in LPS-stimulated RAW 264.7 cells. Treatment with LPS for 6 h dramatically increased the mRNA expression of TNF-α, iNOS, and COX2 (Figure 8). However, 1 h pretreatment with barley sprouts extract before LPS stimulation significantly blocked the expression of mRNA (Figure 8). These results clearly support the inhibitory effect of barley sprouts extract on inflammatory gene expression in the liver of alcohol-treated mice.

## 4. Discussion

In the present study, barley sprouts extract supplementation in the mice fed alcohol for four weeks significantly inhibited the progression of the alcoholic liver injury. Furthermore, the increased levels of the liver injury markers such as hepatic lipid accumulation, as well as serum activities of ALT and AST in alcohol-fed mice were almost completely blocked by treatment with barley sprouts extract. Moreover, its effect on anti-alcoholic liver injury was accompanied by the preservation of hepatic GSH and normalization of the increased serum TNF-α level. This observation suggests that the barley sprouts extract may mechanistically manage the alcohol-induced liver injury.

Accumulating evidence indicates that the role of oxidative stress and inflammation is critical in the pathogenesis of alcohol-induced liver injury [19,20,21]. Chronic exposure to alcohol generates ROS, mainly hydrogen peroxide and superoxide anion in several metabolic steps, which initiate the peroxidation of membrane phospholipids and lipoproteins. The main sources of ROS include the ethanol-inducible cytochrome P450 (CYP) 2E1, aldosterone dehydrogenase (ADH), mitochondrial respiratory chain, iNOS, and peroxisomal β-oxidation of free fatty acids [19]. Moreover, alcohol depletes GSH, which sensitizes the liver to oxidative stress and TNF-α [20]. Meanwhile, alcohol increases the permeability of the intestinal mucosa and subsequently enhances the level of bacterial-derived endotoxin that stimulates Kupffer cells to produce both ROS by nicotinamide adenine dinucleotide phosphate (NADPH) oxidase (NOX) and cytokines [21]. The oxidants generated activate NF-κB in Kupffer cells, which produces of TNF-α and enhances the inflammatory pathway cascade, ultimately leading to tissue injury [19].

GSH is a thiol-containing tripeptide found in high levels, particularly in the liver. It plays a central role in detoxification and serves as a major antioxidant. It is well known that alcohol intake generates excessive ROS and reactive metabolites, accompanied by a profound depletion of hepatic GSH [22]. Consequently, alcohol administration abrogates the balance between antioxidant and oxidant, and oxidative stress is considered as one of the key mechanisms that cause alcohol-induced liver injury. Several studies have focused on the increase in the antioxidant capacity to prevent oxidative liver damage by alcohol. One study showed that the overexpression of SOD, an enzyme that catalyzes the partitioning of superoxide radical, inhibited the lipid accumulation in the liver, whereas deletion of both glutathione peroxidase-1 and catalase promoted alcohol-induced liver injury [23,24,25]. The administration of GSH precursor or antioxidants also reduced the liver injury in alcohol-fed mice by decreasing oxidative stress [26,27,28,29]. In this study, the alcohol-induced reduction in the GSH levels of the liver of the treated mice was significantly prevented by supplementation with barley sprouts extracts. This result indicates that the improvement of the antioxidant capacity in the liver of alcohol-fed mice via maintenance of hepatic GSH by the barley sprouts extract could make it a potentially valuable treatment strategy for alcoholic liver disease. 

The increase in the circulating LPS levels caused by abnormal gut permeability is known to be a key mediator of the inflammatory process in alcoholic liver injury. LPS activates and stimulates Kupffer cells by binding to the CD14 receptor on the cell membrane to release pro-inflammatory cytokines, chemokines, and ROS [19,30,31]. Notably, TNF-α generated in activated Kupffer cells is considered to be one of the most harmful cytokines involved in alcoholic liver injury [30,32]. In the lipid regulatory processes in the body, TNF-α induces lipolysis in adipose tissue, followed eventually by fat accumulation in the liver. Several studies showed that TNF-α causes the release of free fatty acid from adipocytes, and stimulates lipogenesis via sterol regulatory element-binding protein (SREBP)-1c, whereas it inhibits β-oxidation of free fatty acids in the liver [33,34,35,36]. 

In agreement with these reports, the deletion of TNF-receptor 1 (TNFR1) almost completely inhibits the development of alcohol-induced fatty liver [30]. In the present study, chronic alcohol consumption resulted in a significant inflammatory response accompanied by increased circulating TNF-α level, which was significantly inhibited by supplementation with barley sprouts extract. The anti-inflammatory effect of the barley sprouts extract was also demonstrated in the LPS-stimulated RAW 264.7 macrophages. 

## 5. Conclusions

In conclusion, our results indicate that the suppression of TNF-α secretion and maintenance of hepatic GSH by barley sprouts extract contributed to its overall preventive effects against alcohol-induced liver injury.

## Figures and Tables

**Table 1:** Condition for LC-MS/MS analysis of barley sprouts extract.

HPLC Condition		
Column	Luna C_18_ RP column (2.0 × 150 mm, 5 μm)	
Flow rate	0.3 mL/min	
Injection volume	5 μL	
Column temperature	40 °C	
Autosampler temperature	4 °C	
**Mass Condition**		
Ion source		Turbo spray (Negative)
Curtain Gas		10 psi
Collision Gas		N_2_ (Medium)
Ion spray Voltage		−4.2 kV
Source temperature		400 °C
Gas 1		40 psi
Gas 2		50 psi

**Table 2:** Composition of the alcohol liquid diet.

Component	Standard Diet		Alcohol Diet	
	g/L	kcal/L	g/L	kcal/L
Casein	41.4	176.778	41.4	176.778
l-Cystine	0.5	2	0.5	2
dl-Methionine	0.3	1.2	0.3	1.2
Corn oil	8.5	75.14	8.5	75.14
Olive oil	28.4	251.056	28.4	251.056
Safflower oil	2.7	23.868	2.7	23.868
Dextrin maltose	115.2	456.192	24.72	97.89
Choline barbiturate	0.53	0	0.53	0
Fiber	10.0	0	10.0	0
Xanthan gum	3.0	0	3.0	0
mineral	8.75	4.1125	8.75	4.1125
vitamin	2.5	9.5	2.5	9.5
Ethanol	0	0	51.3	358.46
Total energy		1000 kcal/L		1000 kcal/L

Each diet used the vitamin mix at 2.5 g/L diet (g/kg vitamin mix); thiamine HCl, 0.6; riboflavin, 0.6; pyridoxine HCl, 0.7; niacin, 3.0; calcium pantothenate, 1.6; folic acid, 0.2; biotin, 0.02; vitamin B12 (0.1%), 10; vitamin A acetate (500,000 IU/g), 4.8; vitamin D3 (400,000 IU/g), 0.4; vitamin E acetate (500 IU/g), 24.0; menadione sodium bisulfite, 0.08; *p*-amino benzoic acid, 5.0; inositol, 10.00; dextrose 939.0. Each diet also used the mineral mix at 8.75 g/L diet (g/kg mineral mix); calcium phosphate, 500; sodium chloride, 74; potassium citrate, 220; potassium sulfate, 52; magnesium oxide, 24; manganous sulfate, 4.6; ferrous sulfate, 4.95; zinc carbonate, 1.6; cupric carbonate, 0.3; potassium iodate, 0.01; sodium selenite, 0.01; chromium potassium sulfate, 0.55; sodium fluoride, 0.06; sucrose, 117.92.

**Table 3:** List of mouse primer used for real-time reverse transcription-polymerase chain reaction (RT-PCR).

Genes	Primer Sequences	
*TNF-α*	F: GGCCTCTCTACCTTGTTGCC	R: CAGCCTGGTCACCAAATCAG
*IL-1β*	F: TTCACCATGGAATCCGTGTC	R: GTCTTGGCCGAGGACTAAGG
*IL-6*	F: TTGCCTTCTTGGGACTGATG	R: CCACGATTTCCCAGAGAACA
CD14	F: AAACTCGCTCAATCTGTCTTTCACT	R: TCCTATCCAGCCTGTTGTAACTGA
iNOS	F: CGAAACGCTTCACTTCCAA	R: TGAGCCTATATTGCTGTGGCT
COX2	F: GCATTCTTTGCCCAGCACTT	R: AGACCAGGCACCAGACCAAAG
*18S*	F: CAGCCACCCGAGATTGAGCA	R: TAGTAGCGACGGGCGGTGTG
